# Inefficient Placental Virus Replication and Absence of Neonatal Cell-Specific Immunity Upon Sars-CoV-2 Infection During Pregnancy

**DOI:** 10.3389/fimmu.2021.698578

**Published:** 2021-06-03

**Authors:** Ann-Christin Tallarek, Christopher Urbschat, Luis Fonseca Brito, Stephanie Stanelle-Bertram, Susanne Krasemann, Giada Frascaroli, Kristin Thiele, Agnes Wieczorek, Nadine Felber, Marc Lütgehetmann, Udo R. Markert, Kurt Hecher, Wolfram Brune, Felix Stahl, Gülsah Gabriel, Anke Diemert, Petra Clara Arck

**Affiliations:** ^1^ Department of Obstetrics and Fetal Medicine, University Medical Centre Hamburg-Eppendorf, Hamburg, Germany; ^2^ Institute of Clinical Chemistry and Laboratory Medicine, University Medical Center Hamburg-Eppendorf, Hamburg, Germany; ^3^ Heinrich Pette Institute, Leibniz Institute for Experimental Virology, Hamburg, Germany; ^4^ Institute of Neuropathology, University Medical Center Hamburg-Eppendorf, Hamburg, Germany; ^5^ Institute of Medical Microbiology, Virology and Hygiene, University Medical Centre Hamburg-Eppendorf, Hamburg, Germany; ^6^ Placenta Lab, Department of Obstetrics, Jena University Hospital, Jena, Germany

**Keywords:** prenatal infection, vertical transfer, variants of concern, SARS-CoV, T cell response, human trial

## Abstract

Pregnant women have been carefully observed during the COVID-19 pandemic, as the pregnancy-specific immune adaptation is known to increase the risk for infections. Recent evidence indicates that even though most pregnant have a mild or asymptomatic course, a severe course of COVID-19 and a higher risk of progression to diseases have also been described, along with a heightened risk for pregnancy complications. Yet, vertical transmission of the virus is rare and the possibility of placental SARS-CoV-2 infection as a prerequisite for vertical transmission requires further studies. We here assessed the severity of COVID-19 and onset of neonatal infections in an observational study of women infected with SARS-CoV-2 during pregnancy. Our placental analyses showed a paucity of SARS-CoV-2 viral expression *ex vivo* in term placentae under acute infection. No viral placental expression was detectable in convalescent pregnant women. Inoculation of placental explants generated from placentas of non-infected women at birth with SARS-CoV-2 *in vitro* revealed inefficient SARS-CoV-2 replication in different types of placental tissues, which provides a rationale for the low *ex vivo* viral expression. We further detected specific SARS-CoV-2 T cell responses in pregnant women within a few days upon infection, which was undetectable in cord blood. Our present findings confirm that vertical transmission of SARS-CoV-2 is rare, likely due to the inefficient virus replication in placental tissues. Despite the predominantly benign course of infection in most mothers and negligible risk of vertical transmission, continuous vigilance on the consequences of COVID-19 during pregnancy is required, since the maternal immune activation in response to the SARS-CoV2 infection may have long-term consequences for children’s health.

## Introduction

During times of pandemics, individuals with a presumed higher risk for infections compared to the general population instantly receive extensive scientific and clinical attention. This is particularly evident in the context of pregnancy, where pathogens are not only a threat for maternal health, but can also affect pregnancy progression, fetal survival and future children’s health. Candidate examples are prenatal infections with seasonal or pandemic influenza virus strains, which have long been identified to severely affect pregnant women and their unborn children (1–4). Prior to the COVID-19 pandemic commencing in November 2019, the severity and consequences of coronavirus-induced infections have also been thoroughly assessed in pregnant women, e.g. during the epidemic induced by severe acute respiratory syndrome (SARS)-CoV in 2003 (5–8) and the Middle East respiratory syndrome (MERS)-CoV in 2012 (9, 10). Here, both coronaviruses caused significant obstetrical complications, along with severe maternal morbidity and mortality. Furthermore, fetal distress syndromes and enhanced neonatal morbidity and mortality have been reported upon SARS- and MERS-CoV infections (11).

Pregnant women are in a unique immunological state, as rejection of fetal tissue expressing foreign, paternally-derived antigens must be suppressed in order to maintain the pregnancy until term. In brief, this is achieved by a highly tolerogenic immune response (12, 13), e.g. *via* the impaired capacity for antigen presentation, the generation of anti-inflammatory CD4^+^FOXP3^+^ and CD8^+^ CD122^+^ regulatory T cells (14–16) and the suppression of an anti-fetal T effector cell response (17, 18). Pregnancy hormones such as progesterone and glucocorticoids significant contribute to this pregnancy-specific immunological state (19).

The increased pathogen-induced morbidity and mortality observed in the pregnant host has been attributed to this unique immunological status during pregnancy. As shown upon influenza infection in pregnant mice, the impaired capacity for antigen presentation and poor effector T cell response is accompanied by a restricted capacity to mount anti-viral immune responses. The less stringent selective environment even promoted the emergence of influenza virus variants with higher viral pathogenicity (20).

Based on the verification that pregnant women are indeed prone to a severe course of disease when exposed to pathogens such as influenza- and coronaviruses, clinicians and scientists alike immediately focused on pregnant women in the context of the recently emergent coronavirus, referred to as SARS-CoV-2.

Considering the world-wide impact of the SARS-CoV-2 pandemic on morbidity and mortality in infected individuals, it came initially as a surprise that the course of COVID-19 in pregnant women is predominantly benign. These insights arose from more than 30 studies published, encompassing assessments of more than 10.000 pregnant women, as reviewed in (21). A prospective cohort study confirmed low rates of maternal viremia in women with COVID-19 during pregnancy (22) and the regular testing of all in-patient for SARS-CoV-2 revealed that a large percentage of women in labor are asymptomatic for signs of COVID-19, despite a PCR-confirmed positive SARS-CoV-2 nose and throat swab (23, 24).

However, a higher risk of progression to COVID-19 than normal population or non-pregnant women, along with adverse pregnancy outcomes have also been reported in the context of SARS-CoV-2 infection during pregnancy. This is mirrored by an increased risk for admissions to intensive care units, the need for invasive ventilation and higher mortality compared to non-pregnant patients (23, 24). Whether or not these findings can be linked to the emergence of SARS-CoV-2 variants, i.e. B1.1.7, remains to be confirmed in future pregnancy studies. Besides the severe course of COVID-19, pregnancy complications such as premature delivery have been observed.

Moreover, the possibility of vertical SARS-CoV-2 transmission has been put forward. However, opposed to prenatal infections with Cytomegalovirus (CMV), Zika virus (ZIKV) and others, vertical transmission of SARS-CoV-2 has only been reported in rare cases (25–27).

The heterogeneous course of COVID-19 during pregnancy, further complicated by infections occurring at different trimesters, still hinder the thorough evaluation of placental susceptibility to SARS-CoV-2 infection and the consequences for fetal health. Additional insights on the placental response to virus exposure and the possibility of vertical transmission of SARS-CoV-2 are needed in order to understand short- and long-term health disadvantages related to COVID-19 during pregnancy not only for pregnant women, but also their children. A pivotal benchmark for vertical transmission would be that SARS-CoV-2 is capable of infecting and replicating in human placenta cells. We here addressed gaps in knowledge and took advantage of our access to women with prior or acute SARS-CoV-2 infection during pregnancy, in which we assessed maternal and neonatal SARS-CoV-2 viral load in various tissues and also examined the SARS-CoV-2 specific transplacental antibody transfer. Furthermore, we tested whether placental explants, isolated from distinct anatomical regions of SARS-CoV-2 negative women, show signs of viral expression and replication when inoculated with SARS-CoV-2 *in vitro*. Lastly, we evaluated the specific T cell responses against selected structural proteins of SARS-CoV-2 in maternal and cord blood in order to test if the fetus had been exposed to the virus *in utero*.

## Results

### Pregnant Women Mostly Show a Benign Course of COVID-19

Similar to other observations, the majority of women enrolled in our study (n=42) showed a mild or asymptomatic COVID-19 course during pregnancy, irrespective of the trimester in which the infection occurred (**Table 1**, **Supplementary Table 1**). Nevertheless, 12% of the women participating in our study had to be admitted to hospital and 9% needed intensive care. Overall 19% showed a moderate to severe course of disease with oxygen requirement in 3 cases, but no need for mechanical ventilation in any of the cases. This is in line with recent data indicating a higher risk for severe disease in pregnant women compared to the normal population (28). The most frequent symptom was anosmia, present in approx. 62% of the women.

**Table 1:** Demographic details, gestational time point and course of SARS-CoV-2 infection and pregnancy outcome in study participants.

**Demographic details**	
Age (years)	34 (22-45)
Prepregnancy BMI (kg/m^2^)	23,7 (17,9-37,9)
Ethnicity Caucasian	100%
Primigravida	21 (50%)
Comorbidities	
Obesity (BMI >29)	3 (7%)
Hypertension	0
Diabetes mellitus	0
**COVID-19 symptoms**	
Trimester of COVID-19	
First	19%
Second	31%
Third	50%
Asymptomatic	14%
Mild	67%
Moderate	17%
Severe	2%
Fever >38,0°C	29%
Cough	50%
Anosmia	62%
Hospital admittance	5 (12%)
Oxygen supply	3 (7%)
ICU admittance	3 (7%)
Mechanical ventilation	0
**Pregnancy outcome**	
Delivery mode	
Vaginal	22 (52%)
Caesarian section	20 (48%)
Twin pregnancies	4 (9,5%)
Preterm delivery	10 (24%)
<34 weeks	2 (5%)
Birth weight (gram)	3180 (990-4340)
Umbilical artery pH	7,27 (7,09-7,42)
APGAR score 5`<7	0
NICU admission	7 (17%)

^1^Data are presented as median and range, if not stated differently.

With regard to pregnancy complications, we observed that 24% of our study participants delivered preterm, whereby the majority were late preterm deliveries after 34 weeks of gestation. Other authors also reported a higher risk for preterm delivery in pregnant women with COVID-19 (28). Our study included 9.5% twin pregnancies, which are as such prone to preterm delivery and may explain the observed high incidence of preterm births. Two cases with preterm birth had coexisting prenatal complications (Kell intolerance, congenital cystic lung malformation) that were evident before maternal SARS-CoV-2 infection. In one case, a Cesarian section had been scheduled preterm due to fetal growth restriction below the 3rd percentile. COVID-19-infection with mild symptoms occurred at week 20 of gestation. The growth restriction appears to be the result of the uteroplacental insufficiency, mirrored by a high pulsatility index in the uterine Doppler examination and a distinctly elevated soluble FMS-like thyrosin kinase (sFlt-1)-1/placental growth factor (PLGF) ratio at 34 + 6 weeks of gestation. Of special interest is one case where COVID-19 was accompanied by vaginal bleeding, thrombocythemia, elevated liver enzymes and a pathological CTG, which led to preterm delivery at 29 weeks of gestation. Here, the neonate showed signs of moderate fetal distress at birth. The majority of these complication co-incided with the emergence of SARS-CoV-2 mutations, especially the B1.1.7 variant of concern (29), which could be confirmed by sequencing in most of the cases leading to preterm birth (**Supplementary Table 1**). However, since these pregnancies were already at higher risk for e.g. preterm birth (two cases were twin pregnancies) or showed complications already prior to COVID-19, it is difficult to attribute this high incidence of pregnancy complications to a presumably more severe course of COVID-19 upon infection with the B1.1.7 mutation.

### Paucity of SARS-CoV-2 Viral Expression in Term Placenta Upon Prenatal COVID-19

**Figure 1:**
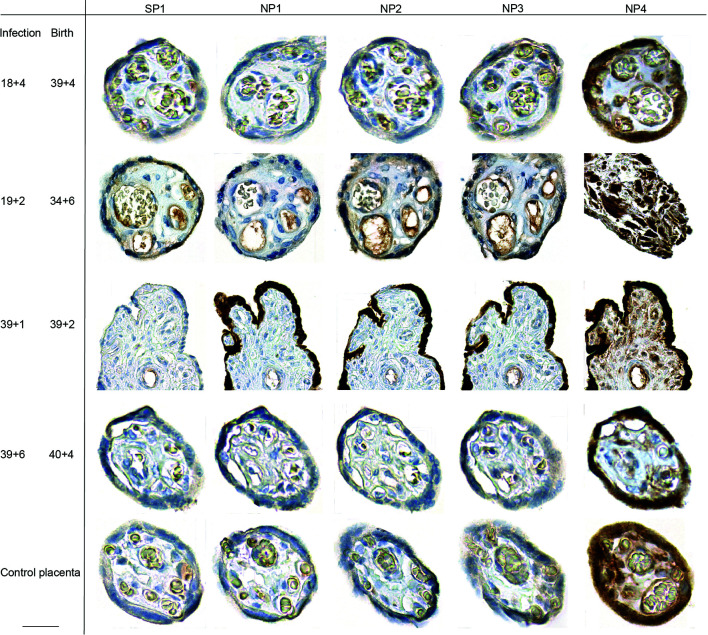
Placental SARS-CoV-2 expression during or upon SARS-CoV-2 infection during pregnancy. SARS-CoV-2 SP and NP expression was assessed by IHC on placental specimen taken at delivery. Time point of infection and delivery is listed at the left side. The antigen targets of the antibodies used for respective IHC, SARS-CoV-2 specific SP or NP, are provided in the top row. Four different antibodies were used to stain for NP, which have been labelled as NP1 to NP4 in top row. One placenta from a non-infected woman was included as control. The third line from the top shows photomicrographs from a positive staining of the syncytiotrophoblast, detected in a placental specimen taken during acute infection (gestation week at infection is 39 + 1 and delivery occurred at 39 + 2). We here deliberately highlight the only positive staining of the sample, additional photomicrographs taken at lower magnification provide a more representative overview of positive staining of the same slide are provided in **Supplementary Figure 1**. Bar inserted at bottom left represents 20 µm.

We assessed the presence of SARS-CoV-2 RNA by RT-qPCR and expression of viral proteins by immunohistochemistry (IHC) of the women affected by COVID-19 during pregnancy in placental tissue taken at term. We used a total of five antibodies for the IHC-based detection of SARS-CoV-2 in placental tissue, targeting the Nucleo- and Spike-Protein (NP, SP; **Supplementary Table 2**). Our antibody repertoire included an antibody that had yielded to positive expression of villous trophoblastic cells in another study (25). Surprisingly, this antibody, labelled as NP4, resulted in an unspecific staining in all placental tissue specimen tested, including placental specimen from non-infected women. The most intense staining could be detected in villous trophoblastic cells, but also in decidual stroma and glands (**Figure 1**). The other antibodies used to detect placental expression of NP and SP by IHC did not yield to a staining pattern that supports SARS-CoV-2 viral presence in the placental specimen in the cases where the SARS-CoV-2 infection dated back weeks of months. We carefully evaluated the potential NP and SP expression in placental samples taken from the cases where delivery occurred under acute infection. Here, in a specimen from one case (#2), a small area of the syncytiotrophoblast showed positive staining of SARS-CoV-2 NP and SP (**Figure 1**), whereas the majority of the tissue from this case (**Supplementary Figure 1**) and all samples from the additional four cases where we performed immunohistochemistry did not reveal any positive SARS-CoV-2 staining by IHC. In this case #2, the asymptomatic woman had been admitted in labor and routine SARS-CoV-2 testing revealed high viral loads in the nasopharyngeal swab. Hence, infection and delivery co-incided within a very short time frame. The lack of or scarce expression of SARS-CoV-2 seen by IHC could be independently confirmed by qPCR, which also did not confirm viral expression except in one of the two samples taken from case #2. We refrained from histopathological analysis of the placental samples due to the small number of cases, the variations with regard to the trimester in which SARS-CoV-2 was contracted, the scarseness of placental infections and the few cases of pregnancy pathologies.

### Inefficient SARS-CoV-2 Replication *In Vitro*

**Figure 2:**
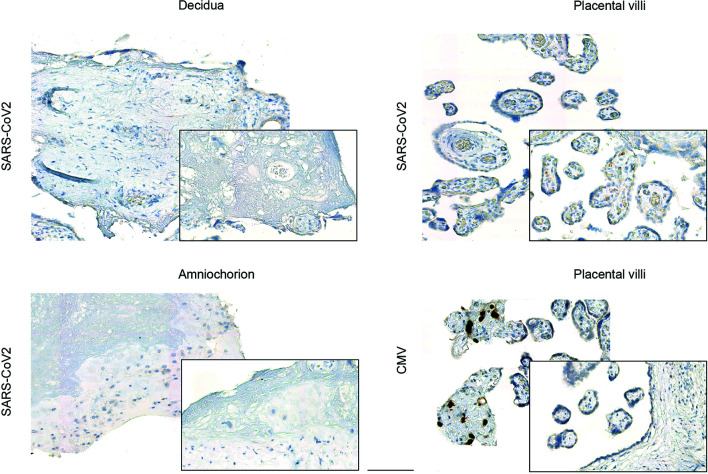
Viral expression in placental explants upon inoculation with human SARS-CoV-2 or CMV isolate. Photomicrographs of placental explants generated from the decidua basalis, the chorionic villi and the amniochorionic membranes were stained for SARS-CoV-2 or CMV expression respectively by IHC, as indicated on left. Inoculation of explants from different anatomical regions of the placenta with a human SARS-CoV-2 isolate did not yield to viral expression, whereas inoculation with CMV confirmed CMV-specific immediate-early antigen expression by IHC predominately in placental villi (bottom left). Bar represents 100 µm.

Next, we used an established *in vitro* infection model of freshly isolated placental explants from women which were negative for SARS-CoV-2. This explant model has previously been utilized, for instance, to confirm Zika virus infection in human placenta (30). We here prepared tissue explants from three distinct anatomical regions: the decidua basalis, which is invaded by the interstitial cytotrophoblast; the chorionic villi, covered with the syncytiotrophoblast layer; and the amniochorionic membranes, which consist of a single layer of cuboidal amnion epithelial cells and is in contact with the fetus *via* the amniotic fluid. Inoculation of the explants with a human SARS-CoV-2 isolate did not result in viral expression, as assessed by IHC (**Figure 2**) in any of the anatomical regions. In addition to the previously demonstrated Zika infection of placental explants, we here also included inoculation with human cytomegalovirus (CMV), which is the most frequently transmitted virus during pregnancy. Here, it was our aim to generate additional evidence that placental explants are susceptible to infection with viral pathogens. Indeed, we could confirm CMV immediate-early antigen expression by IHC upon CMV inoculation of placental explants (**Figure 2**).

**Figure 3:**
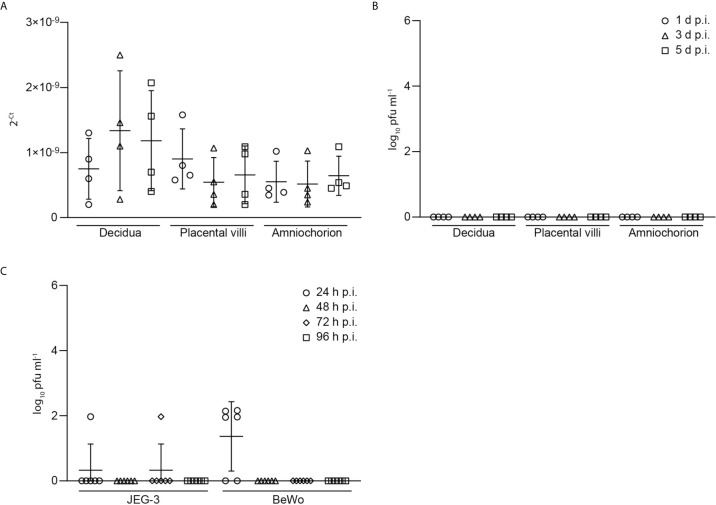
Low levels of viral RNA and viral load in explant supernatants of SARS-CoV-2 inoculated explants. **(A)** Levels of viral RNA, as indicated by Ct-values, was measured by PCR in explant supernatants of SARS-CoV-2 inoculated explants. **(B)** Detection of infectious virus, derived from SARS-CoV-2 plaque test using supernatants of SARS-CoV-2 infected placental explants harvested at 1, 3 and 5 days post infection (d.p.i.). Data are provided as plaque forming units (pfu). **(C)** Infectious virus assessment in supernatants of JEG-3 and BeWo placental cells at 24, 48, 72 and 96 hours post infection (h.p.i.). Lines in scatter plots show median and standard deviation.

Furthermore, we tested the explant supernatants of SARS-CoV-2 inoculated explants by PCR and were only able to detect very low levels of viral RNA as indicated by high Ct-values. No infectious virus could be detected in the supernatants of SARS-CoV-2 infected placental explants (**Figure 3**) further underlining inefficient SARS-CoV-2 replication and lack of production of infectious particles. Additionally, we infected trophoblast cell lines with SARS-CoV-2 to assess the above detected lack in producing progeny virus in independent cell lines of placental origin. In BeWo cells, infectious virus could be detected at 24 h.p.i. but not at later time points (48-96 h.p.i.). In JEG-3 cells, no relevant expression of infectious virus could be detected at all (**Figure 3**). These findings further confirm the inefficient replication capacity of SARS-CoV-2 in placental cells.

### SARS-CoV-2 RNA Detection in Neonates, Cord Blood and Breast Milk

In the samples taken from pregnant women with COVID-19, we also evaluated the presence of SARS-CoV-2 RNA in cord blood and breast milk and could detect viral presence exclusively in breast milk of the woman with the highly acute infection described above, all other tested specimen were PCR negative (**Supplementary Table 1**).

### Anti-SARS-CoV-2 IgG Levels in Maternal and Cord Blood

We further assessed levels of SARS-CoV-2 specific IgG serum levels in women with prenatal COVID-19. As expected, no SARS-CoV-2 specific IgG levels could be detected in women where the infection was still acute, as mounting the anti-SARS-CoV-2 IgG response requires more than two weeks (31). We were surprised to notice very low or even negative anti-SARS-CoV-2 IgG levels in a majority of women where the infection dated back a few months, especially when the course of COVID-19 was mild (**Supplementary Table 2**), which suggests that the humoral immune response mounted against SARS-CoV-2 during pregnancy may wane rather rapidly. IgG levels against SARS-CoV-2 were often higher in cord blood compared to maternal serum. These higher neonatal IgG levels are in line with findings on e.g. influenza, measles and other pathogen-specific IgG at birth, as transplacental IgG transfer from mother to fetus underlies an active, neonatal Fc-receptor-depended transport mechanism rather than a spill-over induced IgG equilibrium between mother and neonate (32).

### SARS-CoV-2 Specific Cell-Mediated Immunity Is Absent in Cord Blood and Lower in Pregnant Compared to Non-Pregnant Women

**Figure 4:**
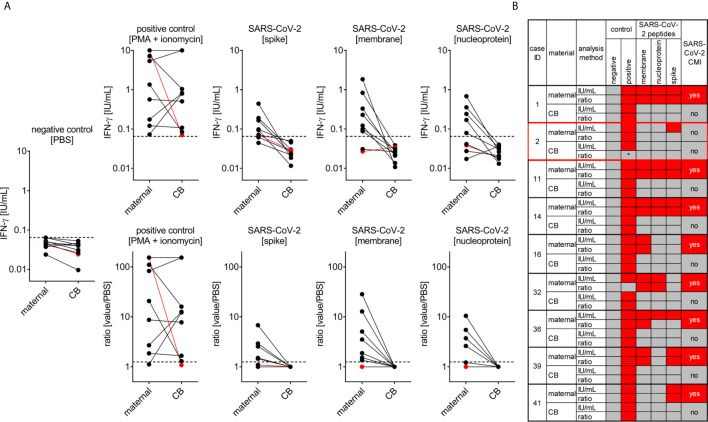
SARS-CoV-2 cell-mediated immunity (CMI) in mother and neonate. Maternal and cord (CB) blood samples of six mother-baby pairs were analyzed for SARS-CoV-2-specific CMI applying an IFN-γ release assay. Blood samples were treated with stimulants as indicated and IFN-ɣ was measured in supernatants. **(A)** Absolute measurements (upper row) and calculated ratios to the negative control (lower row) are depicted as indicated. Red dots refer to the mother-baby pair from case #3, where CMI was tested two days after a positive viral RNA swab. **(B)** Cumulative data interpretation of all measurements applying criteria as outlined in the *Material and Methods* section. *, this individual’s positive control was negative after stimulation with PMA + ionomycin but positive after stimulation with SEB. Clinical details of the participants are listed in **Supplementary Table 1**, please see the case IDs on left to idenitfy the cases in the table.

The T cell response to SARS-CoV-2 infection pivotally determines long-term protective immunity (33). Therefore, we also assessed the SARS-CoV-2 specific cell-mediated immunity in pregnant women and cord blood by applying an interferon gamma (IFN-ɣ) release assay. All tested women of our study (n=9) showed a T-cell response to at least one of the SARS-CoV-2 peptide pools (membrane, NP or SP), except the woman who was enrolled in the study very shortly upon infection (**Figure 4**). The latter observation can be explained by the insufficient time to mount a specific T cell response between infection and blood withdrawal as anti-viral T cell immunity would be expected approximately one week post pathogen exposure.

In contrast to the anti-SARS-CoV-2 T cell response present in maternal blood we could not detect any specific T cell immunity against this pathogen in cord blood obtained from these infected mothers indicating that endogenous neonatal T cells had not been exposed to SARS-CoV-2 antigens (**Figure 4**). This observation is in line with negative testing for viral RNA in neonates, arguing against vertical virus transmission during pregnancy.

Moreover, we detected a stronger a T-cell response at least to the SARS-CoV-2 peptide NP and SP in non-pregnant women upon COVID-19, compared to the response seen in pregnant women, whereby the number of samples tested from non-pregnant women was considerably lower (n = 4) compared to the pregnant group (**Supplementary Figure 2**). The mean age of these non-pregnant women was 30.2 years, the CMI analysis was performed after an average of 53.8 days since COVID-19 diagnosis, when the mean serum SARS-CoV-2 IgG antibody levels were 60.5 AU/ml.

## Discussion

We here report on the outcome of pregnancies affected by maternal SARS-CoV-2 infection at different stages of pregnancy. Albeit relatively small in number of participants, one strength of our study is the broad range of time points at which infections occurred during pregnancy, covering all trimesters. Our findings show that pregnant women show a largely benign course of COVID-19, which is in line with the observations by others, i.e. a study performed in the US, where no evidence of placental infection or definitive vertical transmission of SARS-CoV-2 could be identified (22). Hence, pregnant women are generally not at high risk for a severe course of COVID-19, which is also underpinned by similar levels of respiratory viral load in pregnant and non-pregnant women (22). However, it should be emphasized that pregnant women show higher risk of progression to diseases than normal population or non-pregnant women, which may be explained by the reduced maternal lung volume especially during the third trimester. Furthermore, the emergence of new variants of concern, i.e. B.1.1.7, highlights the importance of continuous vigilance, since such variants may have significant consequences especially for vulnerable groups such as pregnant women. In our study, B.1.1.7 could be confirmed in a few cases, the majority of which also developed pregnancy complications such as preterm birth. However, the causes for preterm birth were heterogeneous and often pre-existing to the infection, which makes it difficult at this point to draw firm conclusions whether or not infection with B.1.1.7 may indeed trigger a more severe course of COVID-19 during pregnancy. Clearly, future studies must focus on this potential imminent threat.

Our findings highlight that a specific SARS-CoV-2 T cell response – which can be detected in maternal blood upon COVID-19 - is absent in cord blood. Previous studies have shown the development of specific T cell responses of the fetus upon congenital viral infection (34, 35). Based on our observation that cord blood T cells do not respond to SARS-CoV-2 peptides, we propose that vertical transmission of SARS-CoV-2 seems unlikely. Whilst one might argue that the uniqueness of the fetal immune environment (36) hampers detection of a specific SARS-CoV-2 response, it appears more likely that we did not detect such specific T cell response in the fetus because SARS-CoV-2 viral antigens did not vertically transfer. In turn, the fetal immune system would not be in direct contact with the SARS-CoV-2 virus, which explains why no fetal SARS-CoV-2 specific T cell response was detectable. Alternatively, other immunological mechanisms may have masked induction and/or detection of fetal anti-SARS-CoV-2 T cell immunity, as seen upon fetal CMV (35) or Plasmodium falciparum exposure (37). It is also conceivable that an intense pro-inflammatory milieu associated with maternal COVID-19 increases placental permeability, which may facilitate vertical transfer of the virus without infecting the placenta. However, given the absence of fetal distress during maternal COVID-19 in our study population and the resistance of the placenta to SARS-CoV-2 infection, it seems highly likely that SARS-CoV-2 does not enter the fetal circulation, at least in our cohort.

The frequency of pregnancy complications is relatively low in our study population, whereas other studies report higher incidences of pregnancy complications. Ethnicity and sociodemographic factors may have contributed to these higher risk for preterm birth or pre-eclampsia seen elsewhere (38–41). Therefore, it will be imperative to assess if COVID-19 is an independent risk factor for pregnancy complications, or results from the higher risk for COVID-19 in certain countries, ethnicities and demographics. Other studies and case reports list premature rupture of membranes, fetal distress, and even fetal death in the context of COVID-19 during pregnancy. Again, it is unknown if these complications were triggered by the infection, or occurred independent of the infection. It should also be noted that some data have been reported prematurely, e.g., pregnancy-related fatalities, and some related publications have been withdrawn by now (42).

The paucity of SARS-CoV-2 viral detection we here observe in placental samples *ex vivo*, which allowed to confirm viral expression exclusively in one sample taken during acute infection, appears to result from the inefficient replication of SARS-CoV-2 in the placenta. Noteworthy, early during the pandemic, the reliable detection of placental infection was challenging, i.e. when it was not yet recognized that certain commercially available antibodies led to unspecific binding and hence, false positive results (25). To date, these challenges have been overcome and the presence especially of viral RNA in placenta, cord blood, vaginal tissue or newborns has been described by a number of studies, along with the introduction of a classification system (43, 44).

SARS-CoV-2 utilizes the canonical cell entry mediators angiotensin-converting enzyme 2 (ACE-2) and the serine protease transmembrane protease serine subtype 2 (TMPRSS-2) for cell entry (45, 46). Placental expression of for ACE-2 and TMPRSS-2 have been assessed in various studies, leading to contradictory findings (46–48). Such variations could result from the different expression of the entry mediators across gestation (23, 24), as not all studies included samples from all trimesters. This notion has recently been confirmed (49), as particularly ACE-2 expression is higher in circumferential villous syncytiotrophoblast early in gestation. The overall low placental ACE-2 and TMPRSS-2 expression may explain why placental infection and vertical transmission of SARS-CoV-2 is generally low (44, 50). Interestingly, in one study investigating cases where mother and neonate where indeed infected with SARS-CoV-2 and placental infection was confirmed, histopathological signs could be detected, i.e. chronic histiocytic intervillositis along with syncytiotrophoblast necrosis. These placental pathologies may either account for the placental infection with SARS-CoV-2 and related maternal-fetal viral transmission, or result from placental infection. However, other studies did not observe specific placental histopathologies in the placental samples expressing SARS-CoV-2 (41, 51).

Also, other cofactors such as Neuropilin-1 can facilitate virus–host cell interactions and enable SARS-CoV-2 cell penetration (52). Neuropilin-1 is expressed in decidual cells and syncytiotrophoblast, but it is reduced in placental samples from pregnancies complicated by pre-eclampsia (52) and fetal growth restriction (53). This would even argue against an increased vertical transfer of SARS-CoV-2 *via* receptor/cofactor-dependent pathways in pregnancies affected by complication. However, vertical transmission of virus may result from mechanical factors, such as the disruption of placental tissue layers, as seen in pregnancy complications.

Our findings on the scarce placental expression of SARS-CoV-2 by PCR and IHC in cases with acute infection suggests that specimen taken from multiple sites of the placenta should be sampled in future studies to allow for a comprehensive evaluation of placental infection during COVID-19 at birth. Such comprehensive evaluation cannot be achieved by testing tissues taken randomly, and multiple sampling will reveal whether the incidence of placental infection is higher than currently postulated. Also, the strategies for SARS-CoV-2 detection must be carefully chosen to avoid false positive expression, such as unspecific staining seen when using certain antibodies, here marked as NP4.

We did not determine SARS-CoV-2 IgM antibodies in umbilical cord blood. Random findings indicate the presence of immunoglobulin (Ig)M and IgG against SARS-CoV-2 antigens in neonates (54). Whilst maternal IgG is regularly transferred across the placenta and can be detected in the neonate/cord blood (55), which we confirmed in our present study, IgM is generally not vertically transferred due to its high molecular weight. Hence, the detection of IgM in neonates at birth has been interpreted as evidence for fetal infection. However, this interpretation should be considered with caution, as the sole detection of SARS-CoV-2-specific IgM does not suffice as evidence for an infection and should be independently confirmed by e.g., PCR or other approaches. In fact, PCR-based testing failed to confirm the presence of SARS-CoV-2 viral RNA in a neonate at birth (28), whereby it must be considered that the virus may have been cleared at the time of testing. Noteworthy, IgM assays are susceptible to false-positive and false-negative results and may therefore be less reliable compared to molecular diagnostic tests based on nucleic acid amplification and detection (16). The scant cord blood IgM detection reported by others may also be interpreted to result from a disruption of the syncytiotrophoblast barrier rather than fetal infection (22).

Taken together, the SARS-CoV-2 pandemic caused a great deal of concern among pregnant women, their attending physician and the society in general. Pregnancy-related COVID-19 concerns initially even went as far as to consider elective termination of pregnancies (56). The inefficient SARS-CoV-2 replication in the placenta provides one explanation for the general protection of the fetus from vertical transmission. However, the generally benign course of COVID-19 in pregnant women – although certainly a great relief for women and physicians alike – comes as a surprise for immunologists. Such unexpected observations hold the great potential to understand how viral challenges affect vulnerable hosts and will certainly foster comparative evaluations of immune responses in response to pathogen challenges, including SARS-CoV, SARS-CoV-2, MERS-CoV, influenza and others. This can be addressed once the SARS-CoV-2 pandemic is under control.

We here report that a robust T cell response against SARS-CoV-2 is mounted by the maternal immune system during pregnancy. Hence, COVID-19 during pregnancy is likely accompanied by maternal immune activation. Such maternal immune activation is known to affect fetal development and impede on long-term offspring’s health, affecting (37, 57, 58) e.g., neurocognitive function and immunity in children later in life. Moreover, since activation and cellular exhaustion has been shown to increase over time in convalescent COVID-19 patients even upon mild courses (59), women infected with SARS-CoV-2 during their reproductive years may experience immune-mediated complications during subsequent pregnancies.

Hence, continuous vigilance is required and women and physicians should not be lulled into a false sense of security by the recent reports on mild courses of COVID-19 for mother and fetus, especially in the light of known or possible emerging variants of concern. Also, maternal immune activation observed in response to the SARS-CoV2 infection may have long-term consequences for children’s health. This is particularly relevant also in the light of the various vaccination strategies, as pregnant women have been excluded from the phase III SARS-CoV-2 vaccination trials and thus, COVID-19 may jeopardize women during pregnancy until herd immunity is achieved.

## Materials and Methods

### Ethical Approval

All study subjects signed informed consent forms and the study protocol was approved by the ethics committee of the Hamburg Chamber of Physicians under the license number PV 7312 and was conducted according to the Declaration of Helsinki for Medical Research involving Human Subjects.

### Biological Sampling

At birth, a venous blood sample was obtained from the mother by peripheral venipuncture. Cord blood was taken from the umbilical cord at childbirth after cord clamping. Placental samples were taken from the decidua, chorionic villi, and amniochorionic membrane and placed in formalin or RNAprotect. Serum samples were stored at -80°C until use and were kept at 4°C after thawing. Breast milk samples were taken upon manual pressure or using a breast pump.

### Quantitative RT-PCR for SARS-CoV-2

Placental samples were stored at 4°C in RNAprotect solution (ThermoFisher; AM7021-500ML). All tissue samples were grinded (Precellys 24, Bertin, Rockville, US) using ceramic beads (Precellys Lysing Kit) and 1ml RNA and DNA free PCR grade water. For RNA/DNA extraction 200μl of the grinded tissue lysate or whole blood was transferred to the MagnaPure96 (Roche, Mannheim, Germany). Automated nucleic acid extraction was performed according to manufacturers’ recommendation with whole process control (Roche control Kit) and final elution volume was 100μl. For virus quantification, a previously published assay was used (60, 61). In brief, the forward primer, 5 ´-ACAGGTACGTTAATAGTTAATAGCmGT-3 (400nM end concentration), 5 ´TATTGCAGCAGTACGCACAmCA-3 ´ (400nM end concentration) and probe 5 ´-Fam-ACACTAGCC/ZEN/ATCCTTACTGCGCTTCG-Iowa Black FQ-3’ (100nM end concentration) were used. Primer and Probes were obtained from Integrated DNA Technologies (IDT, Leuven, Belgium). One-step RT-PCR (25μl volume) was run on the LightCycler480 system (Roche) using one step RNA control kit as master mix (Roche) and 5 μl of eluate. Ct value for the target SARS-CoV-2 RNA (FAM) was determined using second derivative maximum method. For quantification standard in-vitro transcribed RNA (IVT-RNA) of the E gene of SARS-CoV-2 was used. The standard was obtained *via* the European Virus. Linear range of the assay is between 1x10 and 1x10 copies/ml. Quantitative β-globin PCR was performed with commercial TaqMan primer set (Thermo-Fischer, 401846) and Roche DNA control kit. PCR was run on the LightCycler480 system. The amount of DNA was normalized using human DNA standard (KR0454). SARS-CoV-2 RNA levels in tissues were normalized to ß-globin DNA to adjust for differences in tissue input.

### SARS-CoV-2 Immunoassays

Commercially available high-throughput SARS-CoV-2 immunoassays were used for quantitative detection of IgG antibody against SARS-CoV-2 spike protein (Liasion Xl, Diasorine), according to our established protocols (62).

### Assessment of SARS-CoV-2 Specific Cell-Mediated Immunity (CMI)

Overlapping peptide pools of 15-mer sequences covering the whole sequences of the SARS-CoV-2 membrane, and nucleocapsid proteins as well as predicted immunodominant domains of the spike protein (Prot_S) were purchased from Miltenyi Biotec. For the stimulations, 400 µL of heparinized mother whole peripheral blood or cord blood were incubated with either PBS (negative control), 50 ng/mL phorbol-12-myristate-13-acetate (PMA) with 1 µg/mL ionomycin (positive control), 1µg/ml staphylococcal enterotoxin B (SEB, optional additional positive control applied in #6415), or 0.5 µg/mL individual SARS-Cov-2 peptide pools for 24 h at 37°C. The plasma was then collected, diluted with PBS (1:2) and IFN-ɣ release was measured using a fully automated and quality-controlled analyzer (LIAISON^®^ XL, DiaSorin). Samples were considered positive for SARS-CoV-2 CMI if negative control values were below 0.065 IU/mL (lower bound of linearity range) and, for at least one SARS-CoV-2 peptide, stimulation values were above 0.065 IU/mL and the ratio of peptide stimulation to negative control was at least 1.25. Samples with discordant results after peptide stimulation were interpreted as negative. One mother-baby pair that showed strong IFN-ɣ responses after exposure to buffer controls was excluded from the analysis (data not shown). For ratio calculations, values below or above linearity ranges were set to 0.065 IU/mL or 10 IU/mL, respectively.

### Immunohistochemistry to Detect SARS-CoV-2 Spike and Nucleoprotein and CMV in Placental Tissues

All placental tissues (taken *ex vivo* or upon *in vitro* explant culture) were fixed in 4% buffered formalin and processed for paraffin embedding. For the detection of SARS-CoV-2 proteins in *ex vivo* specimen and explants upon *in vitro* culture with SARS-CoV-2, sections were cut at 2 μm and mounted on a glass slide. After dewaxing and inactivation of endogenous peroxidases by 3% hydrogen peroxide, antibody specific antigen retrieval was performed. Anti-SARS-CoV-2 antibodies used in our study are summarized in **Supplementary Table 2**. Specificity of the antibodies for SARS-CoV-2 protein staining in FFPE tissue was already validated on SARS-CoV-2 infected (Hamburg isolate) and non-infected Vero cells that were fixed in formalin and embedded into paraffin (61, 63, 64). One antibody (NP4) was recently used to stain placenta tissue (25) and was also included in our analyses. Immuno-histochemical staining was performed using a Ventana benchmark XT autostainer (Ventana, Tuscon, Arizona, USA). Detection of CMV in placental explants upon *in vitro* infection with CMV was performed following our standard protocol. The monoclonal antibody against CMV immediate early protein 1 and 2 (IE1/2, clone 3H4) was a used (65), which was a generous gift from Thomas Shenk, Princeton University, Princeton, NJ. All slides were scanned using the ZEISS Axio Scan Z1 and ZEN 2.3 (blue edition; Zeiss) software for analysis.

### Preparation and Infection of Placental Tissue Explants

Placental tissue specimens were taken from the amniochorionic membrane, placental villi and maternal decidua within one hour upon delivery by C-section, Sampling was restricted to deliveries by healthy, SARS-CoV-2 and CMV negative mother at term delivery by Cesarean section due to breech position or re-section. A total of n=5 placentae was used to obtain tissue specimen for explant generation. Tissue specimen were washed in PBS with 1% penicillin-streptomycin and 1% gentamycin (Biochrom) and then cut into three small pieces (3-4 mm in diameter) to generate placental explants of each anatomical area for *in vitro* inoculation with SARS-CoV-2 or CMV virus, following a protocol established for Zika infection of placental explants (30). Each of the three explant per region was placed in a separate well of a 48-well plate. The wells had been pre-filled with 500 µl of RPMI medium containing 10% FCS, 1% penicillin–streptomycin, 1% gentamycin. All procedures were carried out under sterile conditions at room temperature.

### Infection of Placental Tissue Explants With SARS-CoV-2

To prepare the inoculum for SARS-CoV-2 infection, the strain SARS-CoV-2/Germany/Hamburg/01/2020 (ENA study PRJEB41216 and sample ERS5312751) was added to RPMI medium containing 10% FCS, 1% penicillin–streptomycin and 1% gentamycin at a concentration of 10^5^ plaque forming units (p.f.u.) in 250 µl per well. As a negative control explants were incubated in 250 µl of the prepared inoculum without virus at 37°C. After 60 min, explants were then be carefully washed three times with PBS and incubated in 500 μl of RPMI medium containing 10% FCS, 1% penicillin–streptomycin and 1% gentamycin at 37°C and 5% CO_2_. Supernatants and tissues were harvested on day 1, 3 and 5 post infection (p.i.). All supernatants were harvested and kept at -80°C until further analysis. The explants of each anatomical region were placed in 4% formalin for at least 48 hours. Formalin-fixed tissue was transferred into ethanol tubes and subsequently embedded into paraffin, following standard protocols. Viral load was determined by viral RNA detection in supernatants using qRT-PCR and plaque test. SARS-CoV-2 tissue expression was assessed by IHC, as described above.

### Infection of Placental Cell Lines With SARS-CoV-2

Placental cell lines JEG-3 (ATCC^®^ HTB-36^™^) and BeWo (ATCC^®^ CCL-98^™^) were used for infection with SARS-CoV-2. Both cell lines were isolated from human choriocarcinoma. They express genes coding for pregnancy hormones, such as human chorionic gonadotropin (hCG), placental lactogen and progesterone. JEG-3 can transform steroid precursors to estrone and estradiol, BeWo expresses genes for estrogen, estrone, estriol and estradiol. JEG-3 or BeWo cells were seeded at 3,5 x 10^5^ cells per ml with 3 ml per well in 6-well plates. At 4-6 hours after seeding, cells were infected with SARS-CoV-2 at a multiplicity of infection (MOI) of 1 in 500 µl Ham’s F-12 medium containing 2% FCS, 1% penicillin–streptomycin and 1% gentamycin for 60 min at 37°C. After washing three times with PBS, 3 ml of infection medium Ham’s F-12 medium containing 2% FCS, 1% penicillin–streptomycin and 1% gentamycin were added and the cells were incubated at 37°C. At the indicated times points 250 µl supernatant per well were harvested and stored at -80°C until determination of viral titer *via* plaque test.

### SARS-CoV-2 Plaque Test

Viral load in supernatants of SARS-CoV-2 infected placental tissue explants and placental cell lines were determined *via* plaque test. VeroE6 cells (ATCC) were seeded in 12 well plates in DMEM containing 10% FBS, 1% penicillin–streptomycin and 1% L-glutamine and incubated at 37°C and 5% CO_2_. At 24 hours after seeding, cells were washed one time with PBS and infected with tenfold dilutions of supernatants diluted in PBS with 150 µl per well and incubated for 30 min at 37°C and 5% CO_2._ During the incubation period, plates were shaken at least every ten minutes. After the incubation, 1,5 ml overlay medium MEM containing 1,25% avicel, 1% penicillin–streptomycin, 1% L-glutamine and 1 µg/ml TPCK-treated Trypsin (Sigma-Aldrich GmbH) were added to each well. After incubation for another 72 hours at 37°C and 5% CO_2_, overlay medium was removed and cells were washed one time with PBS. For fixation, 1 ml 4% paraformaldehyde were added to each well and incubated for at least 30 min at 4°C. Cell layer was stained with crystal violet and plaques were counted.

### SARS-CoV-2 qPCR in Supernatants

Viral RNA was isolated from supernatants using the QIAamp Viral RNA Mini Kit (QIAGEN) according to the manufacturer’s instructions. SARS-CoV-2 RNA levels were then determined by quantitative reverse transcription real-time PCR (qRT-PCR) using the RealStar^®^ SARS-CoV-2 RT-PCR Kit RUO (altona Diagnostics). An internal control provided by the kit was used as a sample preparation control as well as an extended dry spin step for 10 min at 17000g at room temperature.

### Infection of Placental Tissue Explants With CMV

Placental tissue explants were prepared exclusively from CMV-negative women. Two of the three explants per anatomical region were used for infection, the third was used as non-infected control. Explants were incubated overnight with 10^7^ plaque-forming units of human CMV strain TB40E at 37°C and 5% CO_2_ in RPMI medium supplemented with 10% FCS and penicillin, streptomycin, and gentamicin. On the following day, the medium was carefully removed and replaced with fresh medium. Tissue explants were harvested on day 5 and 10 post-infection. CMV immediate-early antigen (IE 1/2)expression was assessed by IHC essentially as described above.

## Data Availability Statement

The original contributions presented in the study are included in the article/**Supplementary Material**. Further inquiries can be directed to the corresponding author.

## Ethics Statement

The studies involving human participants were reviewed and approved by Hamburg Chamber of Physicians, license number PV 7312. The patients/participants provided their written informed consent to participate in this study.

## Author Contributions

A-CT, AD, and PA developed the study and designed most of the experimental set-ups. A-CT enrolled the subjects and collected samples. KH, AD, and A-CT oversaw clinical management of patients. CU, SK, SS-B, AW, NF, KT, and LFB performed most of the analysis, processed the experimental data and designed most of the figures. FS and LFB designed and analyzed SARS-CoV-2 cell mediated immunity. CU coordinated the sample collection, quality control and assessments. KT coordinated biosafety and tissue processing. ML oversaw analyses of SARS-CoV-2 antibody levels in serum samples and viral detection by qPCR in tissues and swabs. AW and NF generated placental explants under the supervision of protocols by URM. GG and SS-B infected placental tissue explants with SARS-CoV-2 virus. GF and WB infected placental tissue explants with CMV. The original draft of the manuscript was written by A-CT and PA and further writing, review and editing were done by all authors. PA supervised the scientific aspects of the project and AD supervised the participant enrolment and ethical aspects. All authors contributed to the article and approved the submitted version.

## Funding

This work was supported by the German Research Foundation (KFO296: AR232/25-2, STA 1549/2-1, DI2103/2-2, KH4617/1-2, BR1730/7-1) to PA, FS, AD, KH, and WB., the Authority for Science, Research and Equality, Hanseatic City of Hamburg (State Research Funding, LFF-FV73) to PA and AD and the *Stiftung für Pathobiochemie und Molekulare Diagnostik* (German Society of Clinical Chemistry and Laboratory Medicine) to FS.

## Conflict of Interest

The authors declare that the research was conducted in the absence of any commercial or financial relationships that could be construed as a potential conflict of interest.
