# Supplementary material for: Inefficient Placental Virus Replication and Absence of Neonatal Cell-Specific Immunity Upon Sars-CoV-2 Infection During Pregnancy
Source: Front Immunol. 2021 Jun 3;12:698578. doi: 10.3389/fimmu.2021.698578 (PMC8211452; doi:10.3389/fimmu.2021.698578)
Supplement: Supplementary file 1 [file DataSheet_1.pdf]

SUPPLEMENTARY MATERIAL

Inefficient placental virus replication and absence of neonatal cell-specific immunity upon Sars-CoV-2 infection during pregnancy

Ann-Christin Tallarek[1], Christopher Urbschat[1], Luis Fonseca Brito[2,3], Stephanie Stanelle-Bertram[3], Susanne Krasemann[4], Giada Frascaroli[3], Kristin Thiele[1], Agnes Wieczorek[1], Nadine Felber[1], Marc Lütgehetmann[5], Udo R. Markert[6], Kurt Hecher[1], Wolfram Brune[3], Felix Stahl[2,3], Gülsah Gabriel[3], Anke Diemert[1,*], Petra Clara Arck[1,*,§]

[1]Department of Obstetrics and Fetal Medicine, University Medical Centre Hamburg-Eppendorf, Hamburg, Germany
[2]Institute of Clinical Chemistry and Laboratory Medicine, University Medical Center Hamburg-Eppendorf, Hamburg, Germany
[3]Heinrich Pette Institute, Leibniz Institute for Experimental Virology, Hamburg, Germany
[4]Institute of Neuropathology, University Medical Center Hamburg-Eppendorf, Hamburg, Germany
[5]Institute of Medical Microbiology, Virology and Hygiene, University Medical Centre Hamburg-Eppendorf, Hamburg, Germany
[6] Placenta Lab, Department of Obstetrics, Jena University Hospital, Jena, Germany
[*] Both authors contributed equally
[§] Correspondence should be addressed to Petra Arck (p.arck@uke.de)

Supplementary Figure 1

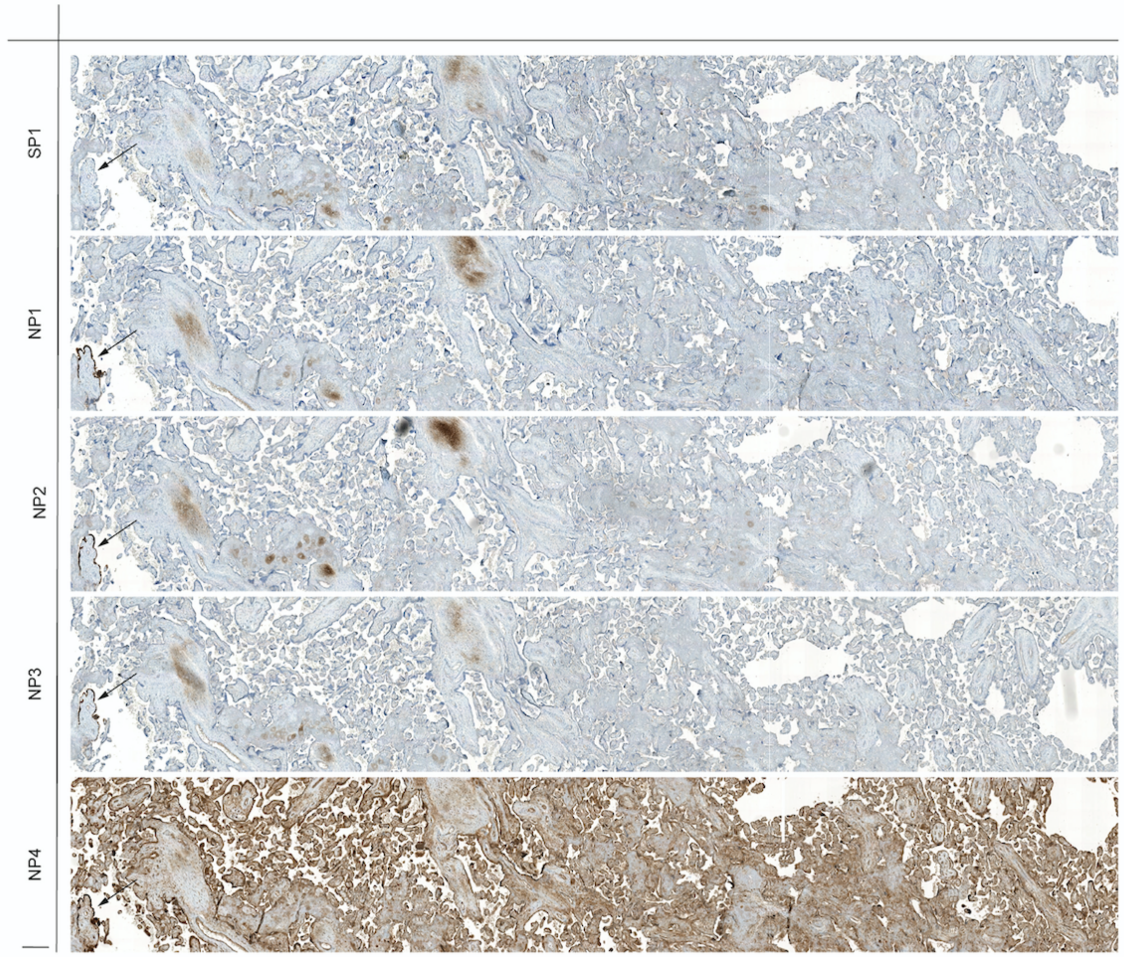

Supplementary Figure 1: Placental SARS-CoV-2 spike (SP) and nucleoprotein (NP) expression, assessed by ICH on placental specimen taken at delivery. All specimen were taken from one specific case of acute infection (case #3, see Supplementary Table 1 for details). Five different commercially available antibodies were used for ICH, as specified in Supplementary Table 2. The photomicrographs show very sporadic SARS-CoV-2 expression in this case, as expression could only be detected in one area of the syncytiotrophoblast, indicated by the arrow in each staining. Note the highly unspecific staining when using the antibody labelled as NP4. Bar represents 200 µm.

Supplementary Figure 2

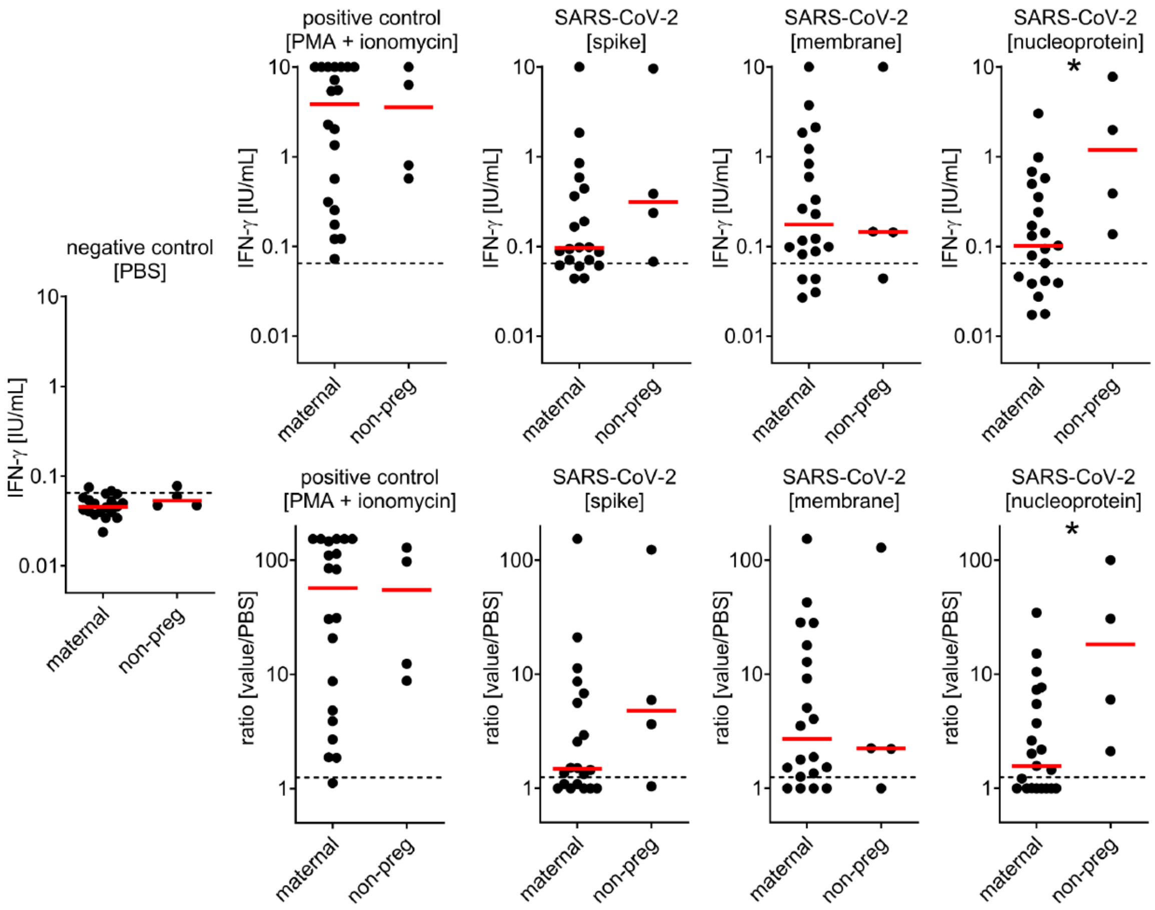

Supplementary Figure 2: Comparison of SARS-CoV-2 cell-mediated immunity (CMI) in pregnant (maternal) and non-pregnant (non-preg) women. SARS-CoV-2-specific CMI was analysed using an interferon gamma (IFN-ɣ) release assay. Blood samples were treated with stimulants as indicated and IFN-ɣ was measured in supernatants. Absolute measurements (upper row) and calculated ratios to the negative control (lower row) are depicted as indicated.

Supplementary Table 1: Covid-19-specific details and pregnancy complications occuring within the studied patient cohort. Cases have been sorted by week of gestation at which SARS-CoV-2 infection was diagnosed

Case ID	Infection (positive PCR, weeks of gestation)	Time interval between infection and delivery (days)	Course of disease	Covid-19 after B1.1.7 emergence, causing at least 50% diseases (Feb 20, 2021) [1]	Pregnancy complications	SARS-CoV-2 status at delivery (variant, if sequenced)	SARS-CoV-2 PCR Placenta	Maternal SARS-CoV-2 IgG antibody at birth (AU/ml)	SARS-CoV-2 mRNA in cord blood	Cord blood SARS-CoV-2 IgG antibody (AU/ml)	SARS-CoV-2 mRNA breast milk	SARS-CoV-2 mRNA neonatal nasopharyngeal swab
1	39+6	5	mild	no	none	positive	negative	negative	negative	negative	negative	negative
2	39+1	1	asymptomatic	no	none	positive	positive/ negative	negative	negative	negative	positive	negative
3	38+5	1	mild	no	none	positive	negative	negative	negative	negative	negative	negative
4	38+1	11	moderate	no	none	negative	negative	negative	negative	negative	negative	negative
5	37+0	17	mild	no	none	positive	negative	negative	negative	negative	negative	not determined
6	36+4	4	mild	yes (B1.1.7 sequenced)	Twins (DC) Preterm birth	positive	negative/ negative	negative	negative/ negative	negative/ negative	not determined	negative/ negative
7	36+3	0	mild	yes (B1.1.7 sequenced)	PPROM Twins (DC) Preterm birth	positive	negative/ negative	negative	negative/ negative	negative/ negative	negative	not determined/ not determined
8	36+2	21	mild	yes (not sequenced)	none	negative	negative	negative	negative	negative	not determined	not determined
9	35+1	33	mild	no	none	negative	negative	127.0	negative	71.6	negative	negative
10	34+6	14	moderate	yes (B1.1.7 sequenced)	Preeclampsia Preterm birth	positive	negative	26.9	negative	negative	negative	not determined
11	33+6	30	moderate	no	none	negative	negative	146.0	negative	92.2	negative	not determined
12	33+4	13	severe	yes (not sequenced)	Preterm birth	positive	negative	37,6	negative	negative	negative	negative
13	32+1	31	asymptomatic	no	PPROM Preterm birth	negative	negative	20.9	negative	negative	negative	not determined
14	30+5	69	mild	no	none	negative	negative	22.6	negative	35,8	negative	not determined
15	29+6	73	mild	no	none	negative	negative	negative	negative	negative	negative	not determined
16	29+3	75	mild	no	none	negative	negative	negative	negative	negative	not determined	not determined
17	28+1	97	mild	no	none	negative	negative	39.4	negative	78.7	negative	not determined
18	28+0	79	mild	no	none	negative	negative	57.7	negative	61.4	negative	not determined

19	27+6	81	mild	no	none	negative	negative	22.1	negative	31.2	negative	not determined
20	27+4	84	mild	no	Congenital anomaly (CCAM)	negative	negative	negative	negative	negative	negative	negative
21	26+4	16	moderate	yes (B1.1.7 sequenced)	Acute placental insufficiency Preterm birth	positive	not determined	3.6	not determined	not determined	negative	negative
22	26+3	98	mild	no	none	negative	negative	not determined	negative	28.0	not determined	not determined
23	24+2	115	mild	no	none	negative	negative	negative	not determined	13.7	negative	not determined
24	24+1	113	mild	no	none	negative	negative	negative	negative	negative	negative	not determined
25	23+3	123	mild	no	none	negative	negative	negative	negative	negative	negative	not determined
26	22+3	114	mild	no	none	negative	negative	negative	negative	negative	negative	not determined
27	21+1	121	mild	no	none	negative	negative	negative	negative	negative	negative	not determined
28	19+2	109	mild	no	IUGR Preterm birth	negative	negative	62.7	negative	57.9	negative	negative
29	19+0	142	asymptomatic	no	none	negative	negative	negative	not determined	negative	negative	not determined
30	18+5	152	mild	no	none	negative	negative	negative	negative	negative	negative	not determined
31	18+4	147	asymptomatic	no	none	negative	negative	negative	negative	not determined	negative	not determined
32	16+4	173	mild	no	none	negative	negative	45.8	negative	57.6	negative	negative
33	14+4	140	mild	no	PPROM Preterm birth	negative	negative	22.7	negative	27.5	negative	not determined
34	14+2	152	mild	no	Twins (DC) IUGR 1st twin Preterm birth	negative	negative/ negative	negative	negative/ negative	negative/ negative	negative	not determined/ not determined
35	13+1	143	moderate	no	Kell-Intolerance Fetal anemia Preterm birth	negative	negative	49,1	negative	49,3	negative	not determined
36	12+5	202	mild	no	none	negative	negative	36.8	negative	65.9	negative	not determined
37	11+6	184	moderate	no	none	negative	negative	65.6	negative	78.9	negative	not determined
38	10+5	185	mild	no	Twins (DC)	negative	negative/not determined	30.2	negative/ not determined	29.9/ 30.3	negative	not determined/ not determined

39	7+5	209	asymptomatic	no	Placenta praevia	negative	negative	negative	negative	not determined	not determined	negative
40	7+4	199	asymptomatic	no	PPROM Preterm birth	negative	negative	negative	not determined	negative	negative	not determined
41	5+1	253	mild	no	none	negative	negative	12.8	negative	22.0	negative	not determined
42	3+0	261	mild	no	none	negative	negative	19.5	negative	27.1	negative	not determined

[1] https://www.hpi-hamburg.de/de/aktuelles/covid-19/daten-der-hamburg-surveillance-plattform/. Abbreviations used in Table: PPROM: Preterm premature rupture of membranes, DC: dichorionic; IUGR: intrauterine growth restriction, CCAM: congenital cystic adenomatoid malformati

Supplementary Table 2: Anti-SARS-CoV-2 antibodies

Antigen	Label*	Clone	Species	Dilution	Order number	Company
Spike	SP1	1A9	Mouse Mab	1:300	#GTX632604	GeneTex
NP	NP1	05	Mouse Mab	1:300	#40143-MM05	Sino Biological
NP	NP2	001	Rabbit Mab	1:5000	#40143-R001	Sino Biological
NP	NP3	n/a	Rabbit PolyAb	1:1000	#40143-T62	Sino Biological
NP	NP4	n/s	Rabbit Mab	1:3000	#A20021	ABclonal

Abbreviations used: Mab: monoclonal antibody; PolyAb: polyclonal antibody; NP: nucleoprotein; n/a: not applicable; n/s: not specified. * refers to column label in Figure 1
